# The Causes of Insanity

**Published:** 1900-03-17

**Authors:** 


### THE CAUSES OF INSANITY.

Speaking at the annual meeting of the Royal Edinburgh Asylum for the Insane, Dr. Clouston made some remarks concerning the causes of insanity which are worthy of note, cutting as they do at the root of the popular modern notion that worry is at the bottom of most of the nervous troubles of the present day. He held, on the contrary, that purely mental and moral causes played a comparatively small part in the production of insanity, as compared with causes which were bodily and physical. In only 11½ per cent. of the cases they had had to deal with had trouble and anxiety or mental shock produced the disease. The remainder of the great mass of the cases were due to causes acting on the brain through the body—drink, faulty development, gross brain disease, strong hereditary predisposition, child bearing, and suchlike causes; and as showing how mental troubles were caused by bodily disease, he said that the recent epidemic of influenza had caused more insanity than all the public and private anxiety in connection with the war. These remarks have a very definite bearing on preventive measures. Without doubt the present generation is apt to coddle its nerves, and almost to plume itself on the delicacy of its organisation. It is widely held that so great is the influence of worry in the production of nerve disease that those prone to nervous breakdown should be in every way protected from irritating and disturbing influences. The medical profession is not entirely without blame in this matter. The phrase, "it is worry, not work, that kills," has received high and wide professional sanction, and it is to be feared that we are far too ready to prescribe a placid mental life rather than a rigid bodily regimen. But we cannot get out of the old philosophical dictum which sums up so much in the few words, *mens sana in*

*corpore sano*, and we find the spirit of the old adage breathing in what Dr. Clouston says about the bodily origin of so much of the insanity which we meet with on every hand. He says that the only two great methods to lessen the nervous disturbances in civilised societies are (1) to live according to physiological and moral law, and (2) to arrange suitable marriages.

